# Supplementary material for: Dopaminergic Projections from the Hypothalamic A11 Nucleus to the Spinal Trigeminal Nucleus Are Involved in Bidirectional Migraine Modulation
Source: Int J Mol Sci. 2023 Nov 28;24(23):16876. doi: 10.3390/ijms242316876 (PMC10706593; doi:10.3390/ijms242316876)
Supplement: Supplementary file 1 [file ijms-24-16876-s001.zip › ijms-2672900-supplementary.pdf]

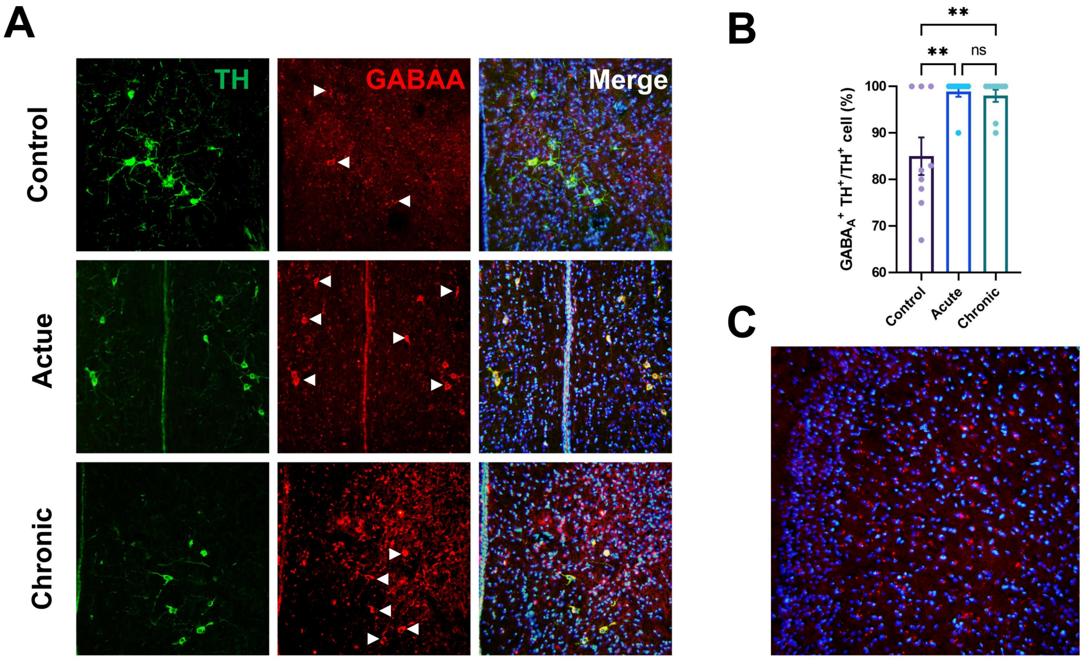

Figure S1. Effect of GTN intervention on the distribution of GABAA receptors in the A11 nucleus. (A) Co-localization of TH+ (Dopaminergic neurons, green) neurons with GABAA receptor+ (red) neurons in the A11 nucleus. (B) Proportion of GABAA+ TH+ neurons to total TH+ neurons in the A11 nucleus. (C) GABAA+ (red) neurons in mouse cerebral cortex. Significance was assessed by one-way ANOVA with post hoc comparison between groups (**$P < 0.01$). All data are presented as the mean ± S.E.M.
